# Factors Influencing medical students’ attitudes toward mental illness

**DOI:** 10.1192/j.eurpsy.2026.11970

**Published:** 2026-07-31

**Authors:** G. Chakchouk, N. Regaieg, F. Guermazi, Y. Trabelsi, I. Feki, J. Masmoudi, I. Baati

**Affiliations:** Department of Psychiatry A, Hedi Chaker Hospital, Sfax, Tunisia

## Abstract

**Introduction:**

Demographic and experiential factors influence attitudes towards mental illness in medical students.

**Objectives:**

This study explores variations in attitudes toward Mental Illness according to gender, academic factors, and contact with mental health among medical students.

**Methods:**

A cross-sectional analytical study was conducted among students in the faculty of medicine of sfax. Participants completed an online questionnaire comprising of socio-demographic data, personal and family history, academic factors and the Community Attitudes toward the Mentally Ill (CAMI) scale.

**Results:**

CAMI total scores showed significant variations across several factors. Scores differed by year of study (p = 0.006), with the highest mean rank among second-year students. Male students had higher scores than females (p = 0.002). Students who had consulted a mental health professional, those with a personal psychiatric diagnosis, or those who had friends with psychiatric history exhibited lower CAMI scores, reflecting more positive attitudes toward mental illness (p = 0.016, p = 0.042, and p = 0.024, respectively).

On the Authoritarianism subscale, scores varied significantly by academic year (p = 0.011), with higher values among first- and second-year students compared to fourth-year students. Male students expressed more authoritarian attitudes (p < 0.001), while those who had consulted a professional or had a psychiatric diagnosis showed less authoritarianism (p = 0.014 and p = 0.047, respectively) Students who actively participated during the exams scored lower on Authoritarianism subscale (p=0.007).

Benevolence increased significantly with academic progression (p = 0.001) and was higher among students who had previously consulted a mental health professional (p = 0.025). An interesting finding is that the students who actively participated in the medical or psychiatric examination of patients had higher benevolence scores (p=0.019).

For Social Restrictiveness, male students were more socially restrictive (p = 0.003), whereas those with a psychiatric diagnosis demonstrated less restrictive attitudes (p = 0.043).

**Conclusions:**

Students’ attitudes toward mental illness varied by gender, academic year, and prior mental health experiences.

This suggests that active and experiential learning opportunities are more affective at reducing stigmatizing beliefs than passive curricular exposure, providing therefore new targets for anti-stigma interventions.

**Disclosure of Interest:**

None Declared

